# *Scedosporium apiospermum* lung disease in a patient with nontuberculous mycobacteria

**DOI:** 10.1002/rcr2.691

**Published:** 2020-12-10

**Authors:** Hiroaki Ogata, Eiji Harada, Isamu Okamoto

**Affiliations:** ^1^ Research Institute for Diseases of the Chest Graduate School of Medical Sciences, Kyushu University Fukuoka Japan; ^2^ Department of Respiratory Medicine National Hospital Organization Fukuoka National Hospital Fukuoka Japan

**Keywords:** Haemoptysis, nontuberculous mycobacteria, pulmonary mycetoma, *Scedosporium apiospermum*, voriconazole

## Abstract

Although tuberculosis is a major underlying cause of pulmonary mycetoma due to *Scedosporium apiospermum*, little is known about coinfection with nontuberculous mycobacteria (NTM) and *S. apiospermum*. A 67‐year‐old man with NTM presented with recurrent haemoptysis. Computed tomography of the chest revealed pulmonary mycetoma in the left upper lobe of the lung, and culture of bronchial washing fluid yielded *S. apiospermum*. Oral voriconazole therapy ameliorated both haemoptysis and mycetoma findings. As far as we are aware, this is the first reported case of *S. apiospermum* lung disease in a patient with NTM but without tuberculosis. The possibility of *S. apiospermum* infection should thus be considered in the differential diagnosis of pulmonary mycetoma. Although *S. apiospermum* mycetoma resembles aspergilloma, antifungal strategies for these two conditions differ, with the collection of culture specimens such as by bronchoscopy being compulsory for accurate diagnosis and appropriate management of *S. apiospermum* infection.

## Graphical abstract

Although tuberculosis has been identified as a major underlying cause of pulmonary mycetoma due to *Scedosporium apiospermum* infection, little is known about coinfection with nontuberculous mycobacteria (NTM) and *S*. *apiospermum*. We now report a case of *S*. *apiospermum* lung disease in a patient with NTM infection, but with neither tuberculosis nor an immunosuppressed condition. This case reinforces the fact that clinicians should be aware of the possibility of *S*. *apiospermum* infection even in patients who are immunocompetent or free from tuberculosis.

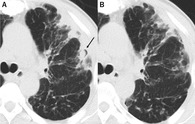

## Introduction

*Scedosporium apiospermum* is a widely distributed saprophytic fungus. It infects the lungs only rarely, with pulmonary manifestations ranging from simple colonization to mycetoma formation and invasive disease [1]. Clinical and radiological features of pulmonary *S. apiospermum* infection are similar to those of disease caused by *Aspergillus* spp., but it is essential that these two fungal infections be distinguished to allow for appropriate treatment, given the differences in antifungal resistance between *Scedosporium* and *Aspergillus* spp. [2].

Tuberculosis has been identified as a major predisposing factor for pulmonary *S. apiospermum* mycetoma [1]. Although aspergilloma develops readily in individuals infected with nontuberculous mycobacteria (NTM) as well as in those with tuberculosis, little is known about the relation of *S. apiospermum* mycetoma to NTM infection. We now report a case of haemoptysis due to fungus balls caused by *S. apiospermum* in a patient with NTM‐related lung disease.

## Case Report

A 67‐year‐old Japanese man presented at our hospital with recurrent haemoptysis that he had experienced for 10 months. He had been undergoing management for NTM lung disease due to *Mycobacterium kansasii* and *Mycobacterium avium*—including the administration of several antibiotics (such as rifampicin, ethambutol, clarithromycin, and sitafloxacin)—for more than 15 years. He thus initially received successful antibiotic treatment over two years for *M. kansasii* lung disease. Five years later, he developed *M. avium* lung disease. Administration of antibiotic therapy over another two years ameliorated the disease, but it worsened again six months after the end of the therapy. Despite continuous antibiotic treatment for the past six years, the NTM lesions had grown slowly. Three years before presentation at our hospital, he also underwent segmentectomy as a diagnostic treatment for a tumour‐mimicking nodule in the right upper lobe of the lung, and the resected specimen revealed massive caseous necrosis with *M. avium* pathogens. He had no other history of serious infection, immunodeficiency disorder, or taking of immunosuppressive medication, and he had a negative human immunodeficiency virus serostatus and a normal glycated haemoglobin concentration. He was a 4.5 pack‐year former smoker, but had refrained from smoking for 42 years. His occupation had been a steelworker until he retired eight years before admission.

**Figure 1:**
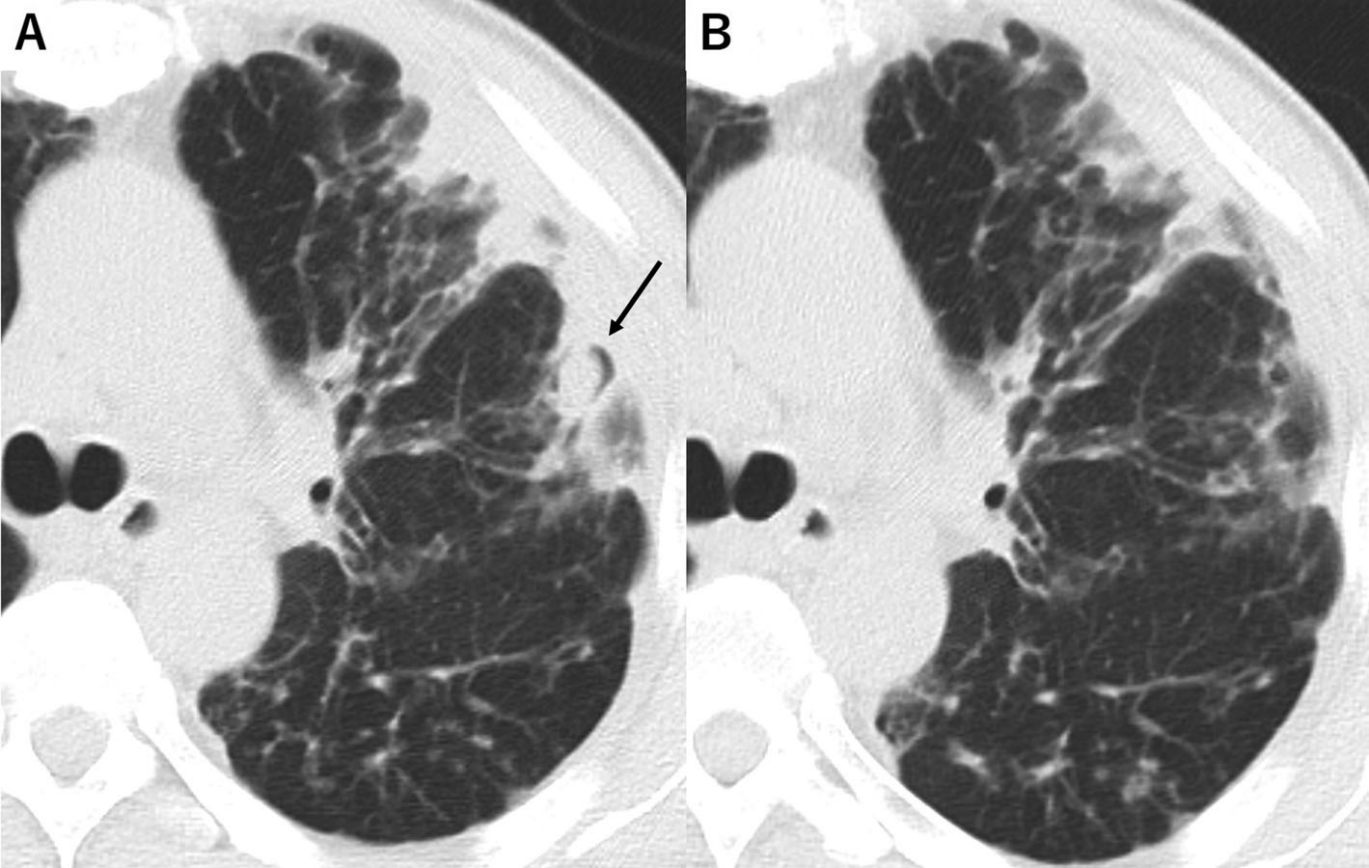
Computed tomography findings of the chest. (A) Cavitary lesions with fungus balls (arrow) were detected in the left upper lobe of the lung on admission. (B) Treatment with voriconazole for six months ameliorated the cavitary lesions.

Physical examination revealed that the patient was afebrile and had an oxygen saturation of 97% on room air. There were no abnormal breath sounds. Blood analysis showed an elevated serum level of C‐reactive protein (3.71 mg/dL). An interferon‐γ release assay for tuberculosis, β‐d‐glucan assay, and *Aspergillus* galactomannan enzyme‐linked immunosorbent assay were all negative. Computed tomography (CT) of the chest revealed cavitary lesions with intracavitary nodules, with the typical appearance of fungus balls, in the left upper lobe of the lung (Fig. 1A). No pathogens other than NTM were detected by Gram staining, acid‐fast staining, or culture of sputum.

**Figure 2:**
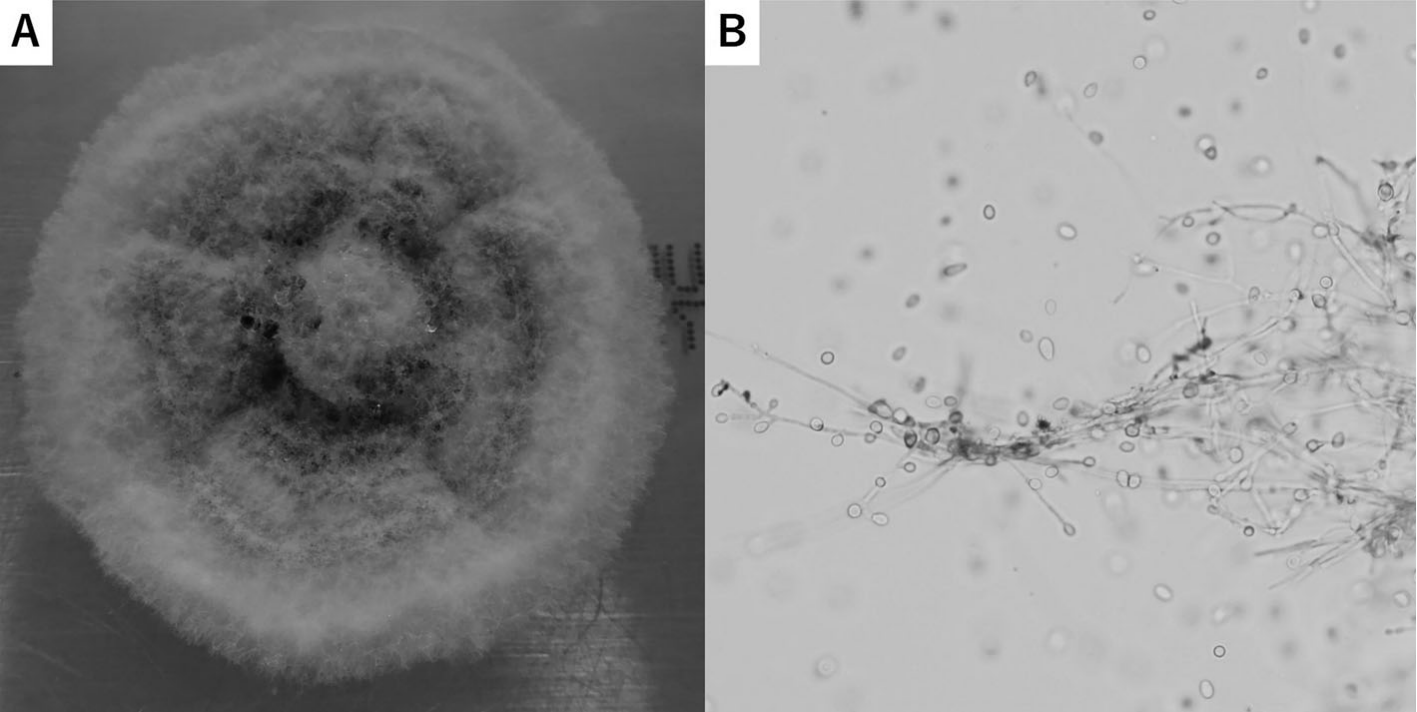
*Scedosporium apiospermum* cultured from bronchial washing fluid collected from the left superior lobar bronchus. (A) Macroscopic findings for culture on Sabouraud dextrose agar. (B) Lactophenol cotton blue staining (original magnification: 400×).

Bronchoscopy revealed purulent secretion throughout the trachea and bronchi. Culture of bronchial washing fluid from the left superior lobar bronchus yielded *S. apiospermum* (Fig. 2), leading to a diagnosis of pulmonary *S. apiospermum* mycetoma. Oral treatment with voriconazole was initiated at a dose of 400 mg/day, which was subsequently increased to 500 mg/day on the basis of therapeutic drug monitoring. Taking into account the interactions between rifampicin and voriconazole, we stopped rifampicin before starting voriconazole therapy. The frequency of haemoptysis declined, and the cavitations and fungus balls in the CT scan were ameliorated at six months after the initiation of the antifungal agent (Fig. 1B).

## Discussion

*Scedosporium apiospermum* is a rare fungal cause of pulmonary infection. A literature review revealed the presence of cavitary lesions containing fungus balls, often referred to as pulmonary mycetoma, in 24.3% of reported cases of pulmonary *S. apiospermum* infection. It also indicated that pulmonary mycetoma due to *S. apiospermum* occurred predominantly in individuals with tuberculosis or in those with an immunocompromised condition such as is associated with solid organ transplantation, haematological malignancy, or chronic corticosteroid use [1]. Pulmonary mycetoma due to *Aspergillus* spp. infection, or aspergilloma, is frequently accompanied by either tuberculosis or NTM infection. However, in contrast to tuberculosis, there have been only two reported cases of coinfection with *S. apiospermum* and NTM in the lung, both of which were in patients with a history of tuberculosis [3, 4]. Although the reason for the rareness of coinfection with NTM and *S. apiospermum* is unknown, it might be at least in part due to the lower frequency of cavitations in individuals with NTM than in those with tuberculosis [5]. In addition, patients with cavitary NTM pulmonary disease were found to be less likely to have underlying comorbidities than were those with cavitary tuberculosis [5], which might influence the tendency to develop *S. apiospermum* coinfection. On the other hand, an in vitro study showed that *M. avium* infection attenuated lymphocyte immune responses via the programmed cell death‐1 pathway, which might be expected to facilitate the development of *S. apiospermum* infection [6]. As far as we are aware, the present case is the first reported for *S. apiospermum* lung disease in a patient with NTM infection, but with neither tuberculous infection nor an immunosuppressed condition.

As in the present case, haemoptysis is reported to be a common and potentially life‐threatening symptom of pulmonary *S. apiospermum* mycetoma, as it is also in pulmonary aspergilloma. In addition, the radiological findings of pulmonary *S. apiospermum* mycetoma mimic those of aspergilloma [1]. Although it is difficult to distinguish between these two conditions, a definitive diagnosis of scedosporiosis is important for clinical management because it presents unique treatment challenges, with *S. apiospermum* being resistant to several antifungal agents including amphotericin B [2]. Given that aspergilloma has been identified as a risk factor for haemoptysis among patients with mycobacterial disease [7], *S. apiospermum* infection may have a similar unfavourable effect on the development of haemoptysis in this population.

Voriconazole is the first‐line antifungal agent for the treatment of scedosporiosis [2]. However, the required duration of this therapy is unknown [1, 2]. The present patient was not a candidate for surgical treatment because of his extensive NTM lung disease, with careful monitoring of such patients thus being necessary.

With regard to the differential diagnosis of pulmonary mycetoma, clinicians should be aware of the possibility of *S. apiospermum* infection even in patients who are immunocompetent or free from tuberculosis. The increasing prevalence of NTM infection worldwide will likely result in an increase in the number of cases of simultaneous respiratory infection with *S. apiospermum* and NTM. Given that pathogen identification is critical for accurate diagnosis of *S. apiospermum* infection, the collection of specimens for culture such as by bronchoscopy is necessary for the appropriate management and treatment of this disease.

### Disclosure Statement

Appropriate written informed consent was obtained for publication of this case report and accompanying images.
